# Improving the quality of antibiotic prescribing through an educational intervention delivered through the out-of-hours general practice service in Ireland

**DOI:** 10.1080/13814788.2020.1784137

**Published:** 2020-08-06

**Authors:** Nuala O’Connor, Roisin Breen, Mícheál Carton, Ina Mc Grath, Norma Deasy, Claire Collins, Akke Vellinga

**Affiliations:** a Elmwood Medical Practice, Frankfield, Cork, Ireland; b ICGP Irish College of General Practitioners, Dublin, Ireland; c Antimicrobial Resistance and Infection Control Divison, HPSC Health Protection and Surveillance Centre, Dublin, Ireland; d Quality Improvement Team, Health Service Executive, Dr Steevens Hospital, Dublin, Ireland; e Therapy Unit, Cork Kerry Community Healthcare, Listowel, County Kerry, Ireland; f Communications Division, Health Service Executive, Model Farm Road Business Park, Cork, Ireland; g School of Medicine, National University of Ireland, Galway, Ireland

**Keywords:** Antibiotics, co-amoxyclav, out-of-hours, quality improvement, general practice

## Abstract

**Background:**

Antibiotic resistance is a threat to our health and health systems. Up to 70% of antibiotics are prescribed in general practice. In Ireland, Out-of-hours (OOH) services are mostly provided by co-operatives of GPs and the 11 main OOH centres cover up to 90% of the population. More than 80% of GPs are involved in OOH care in their area, which provides an opportunity to deliver education and awareness through this centralised system.

**Objectives:**

To analyse the change in the quality of antibiotic prescribing after the introduction of an educational intervention categorising antibiotics into a red (avoid) and green (preferred) panel.

**Methods:**

Educational information for the GP was developed based on the national prescribing guidelines. A particular focus was to reduce co-amoxyclav prescribing. An electronic pop-up message to record whether an antibiotic was prescribed, was displayed at the end of each consultation in the patient management software of the OOH-centre, after the decision of prescribing was made. Antibiotic prescribing was compared for a 13-week period (week 47–week 7) in 2016/2017 with 2017/2018.

**Results:**

Pre-intervention prescribing of red antibiotics was 44% which reduced to 17% after the intervention. The mean percentage of co-amoxyclav, the most prescribed non-firstline prescription, was 33% of all antibiotic prescriptions which dropped to 10%.

**Conclusion:**

Our intervention implemented in the OOH GP service categorised antibiotics into red prescriptions and green (firstline) prescriptions, which was recorded through an electronic pop-up message, resulted in an absolute reduction of 27% in red prescriptions and more than 23% in co-amoxyclav prescriptions.

KEY MESSAGES

Through simple and easy to understand categorisation of antibiotics into red (to be avoided) and green (firstline/preferred), important quality improvements can be made.
As most GPs provide regular Out-Of-Hours service, the delivery of this intervention through this setting has the potential to reach many more GPs compared to daily practice.

## Introduction

Antibiotic resistance is a major health problem that is caused mainly by antibiotic consumption [1,2]. The highest volumes of antibiotics are prescribed by general practitioners (GPs) [3]. Multifaceted interventions have shown to be useful in improving antimicrobial prescribing, in particular when addressing behavioural change. However, most interventions focus on GPs in daily clinical practice, while other settings may also provide opportunities [4].

Whereas continuous care is generally provided by the GP, out-of-hours (OOH) services are mostly provided by co-operatives of GPs who may not be the patient’s regular GP. In Ireland there are 11 OOH centres, covering up to 90% of the population. Most GPs (more than 80%) are involved in the OOH care in their area [5]. This offers a unique opportunity to deliver education and awareness to many GPs through this centralised system as opposed to a more fragmented practice by practice approach. By targeting OOH centres, the intervention can be delivered to many more GPs with relatively fewer resources and a potentially larger impact.

According to the yearly surveillance reports from the European Centre for Disease Prevention and Control (ECDC), Ireland’s community antimicrobial consumption of 22.9 daily defined doses (DDD) per 1000 inhabitants per day, is above the European average (21.8 DDD), with the Netherlands having the lowest consumption (10.1) [6]. The broad-spectrum antibiotic, co-amoxyclav, is the most frequently prescribed antibiotic in Ireland [7] and the fifth highest in Europe (13.0 versus European average of 11.5 DD per 1000 inhabitants per day) [6].

Up to 25% of the OOH consultations are patients presenting with infectious diseases [8,9], in particular respiratory infections (20% of consultations) [10,11]. Most of these consultations for an infection result in an antibiotic prescription, which makes that OOH prescriptions are more often antibiotics than general GP consultations [11]. As many of these consultations for infections could be dealt with outside OOH, patient education to improve knowledge on how to manage symptoms and expectations of recovery could reduce inappropriate consultations and subsequent inappropriate antibiotic prescribing.

This paper describes the delivery of an intervention to improve the quality of antibiotic prescribing through education and support for GPs and patients using the OOH setting as a vehicle to increase the number of GPs reached.

## Methods

### Study design

Observational quality improvement study.

### Setting: OOH service

For this (feasibility) study, two OOH services were approached to participate: Cork city (south of Ireland) and Killarney (south-west Ireland). A total of 240 GPs participate in providing OOH service in this area. The choice to start and test the intervention with these two OOH services was due to the availability of a database manager in this area.

### Intervention development

From November 2016 to February 2017, an inventory was made of the tools and information available to the project to support and evaluate an intervention to address the quality and quantity of prescribing. This provided the following:

- OOH services have bespoke software systems, which combine usual patient management information systems with a more agile interface for service needs in OOH. The software includes the option to prescribe (and query) antibiotics. However, only 10% of prescriptions in OOH were written using the software and most information is provided in the free text fields.
- National antibiotic prescribing guidelines provide detailed information on when and what antibiotics to prescribe according to patient group and condition (www.antibioticprescribing.ie).
- The Health Service Executive (HSE), Ireland’s National Health Service, runs a yearly public education campaign on the appropriate use of antibiotics (www.undertheweather.ie). This information is aimed at discouraging antibiotics for self-limiting infections and encourage self-help. The information is freely available online and leaflets are provided to all GP and OOH services.
- The ‘undertheweather.ie’ website aims to:
  ○Provide advice to the public on how to mind themselves or a loved one through a range of common illnesses, such as colds and flu, tummy bugs, ear infections, rashes and more.
  ○Provide basic health knowledge, and help keep people from making unnecessary and expensive visits to GP surgeries and emergency departments.
  ○Reinforce the message that you do not need an antibiotic to recover from colds, flu or a range of common illnesses.

An electronic pop-up message was displayed at the end of each consultation in the patient management software, after the decision of prescribing was made. The pop-up asked whether an antibiotic was prescribed. If answered ‘yes’, a new window opened in which the GP ticked which antibiotic was prescribed.

Improvements were made after an initial trial cycle, including:

- Back button if an invalid yes was answered in the pop-up message ‘Have you prescribed an antibiotic’.
- Changed pop-up message to ‘Have you prescribed an oral antibiotic’ to exclude topical prescribing.
- Additional information on generic antibiotic names.

The intervention was developed based on the national prescribing guidelines and firstline antibiotics were categorised into a green panel with ‘preferred antibiotics in primary care’ according to the three main infections and a red panel with ‘antibiotics to be avoided in primary care’, with specific indication for when to use these antibiotics (Table 1).

**Table 1.** Green (preferred) and red (to be avoided) antibiotics in primary care.

**Green (preferred) antibiotics**	
Respiratory tract infections (upper and lower)	Penicillin V, Amoxicillin, Doxycycline, Amoxicillin & Clarithromycin if Community Acquired Pneumonia, Clarithromycin if true penicillin allergy/specific indication
Urinary tract infections	Trimethoprim, Nitrofurantoin, Fosfomycin, Cephalexin
Soft tissue infections	Flucoxacillin, Doxycycline, Lymecycline, Trimethoprim
**Red (to be avoided) antibiotics**	
Co-amoxyclav (unless animal or human bite, facial cellulitis, post-partum endometritis, caesarean wound infections, pyelonephritis)	
Ciprofloxacin (unless proven resistant UTI or acute prostatitis)	
Most third generation Cephalosporins	
Clindamycin	
Azithromycin (only on advice consultant or if treating STI)	
Moxifloxacin (only on advice consultant)	
Macrolides (unless true penicillin allergy/specific indication e.g. mycoplasma, helicobacter eradication)	

**Figure 1.**
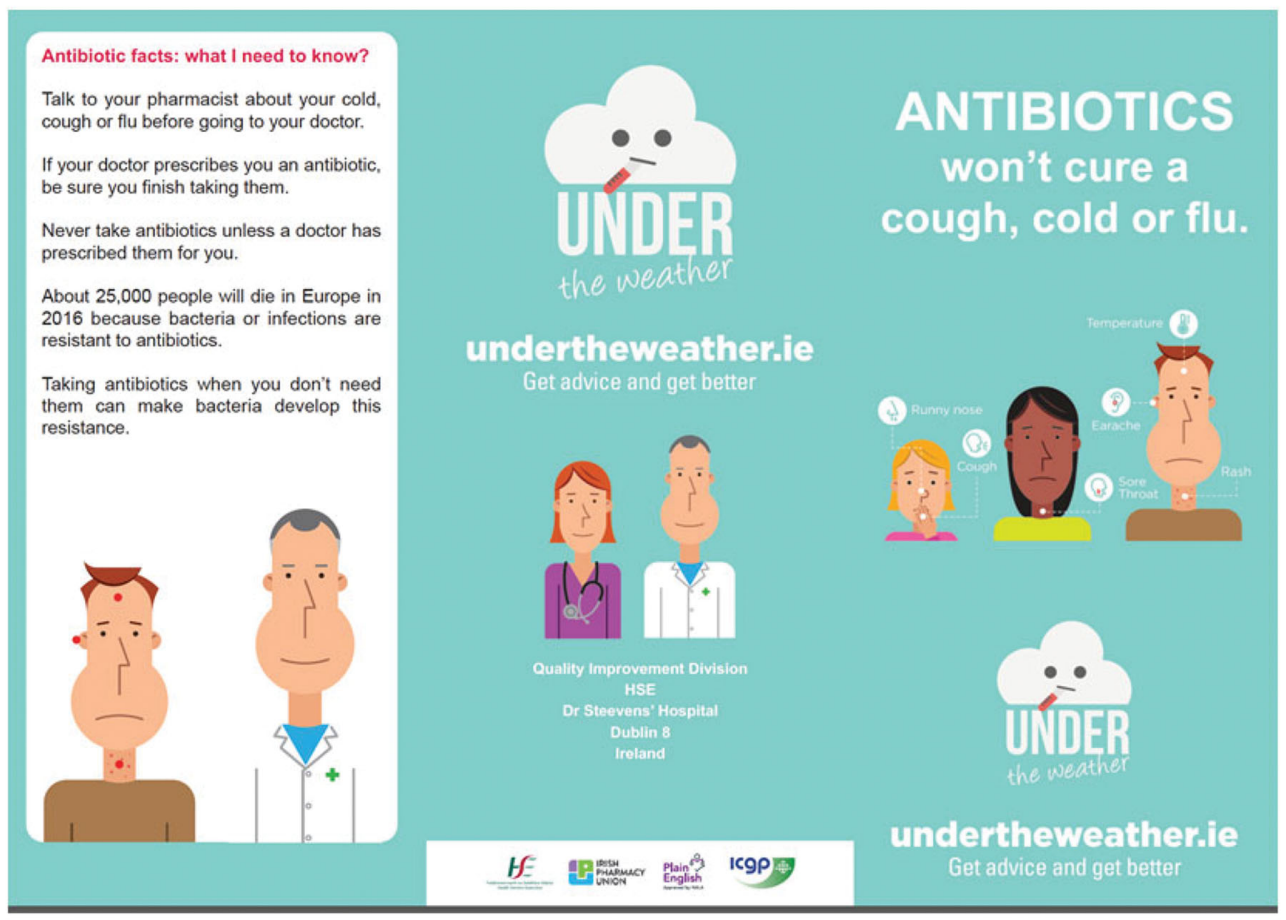
Patient education leaflet based on the ‘undertheweather’ campaign.

Supports were developed to help GPs improve their prescribing. The supports were based on the ‘undertheweather’ website. These included leaflets, stickers for younger patients, waiting room leaflets and posters and mouse mats with the Green and Red antibiotics (Figure 1).

To endorse and engage GPs in the intervention, automated educational text messages were sent to participating GPs upon prescription of red antibiotics, erythromycin (particularly discouraged due to high levels of resistance), or clarithromycin (in relation to penicillin allergy).

### Data collection – General

The intervention took place from week 47 (20 November 2017) to week 7 (18 February 2018). The study was launched by the extraction of data to provide baseline information (week 47, 2017–week 7, 2018). Over a period of 13 weeks, the percentage of red (avoid), green (preferred) and co-amoxyclav prescriptions was calculated based on the total number of antibiotics prescribed during this period.

The objective was to analyse the change in the quality of antibiotic prescribing after the introduction of an educational intervention categorising antibiotics into a red (avoid) and green (preferred) panel. The pop-up message in the OOH software system would act as a nudge for the prescriber while meanwhile providing a method for data collection.

### Data collection – Baseline

To allow evaluation of the impact of the intervention, a baseline prescribing prevalence was established based on retrospective OOH electronic prescription and consultation notes analysis. Baseline prescribing data were extracted from the patient management software. As only 10% of the prescriptions were generated through this software, an additional data extraction was set up to include the free text consultation information of 1000 consultations from November of 2016 to February 2017. The 1000 consultations were analysed based with standard search terms to query the free text of all the consultations (including antibiotic, commercial names of antibiotics, antibiotic group names). The search terms were subsequently categorised to identify green or red antibiotic prescriptions.

### Data collection and feedback during the intervention

Once the pop-up message was integrated into the OOH patient management system (November 2017), data was directly extracted and included in an automated report. After consultation with the participating GPs, the report allowed generation of an overview of each key measure as a run chart. Key measures were the number/percentage of co-amoxyclav and/or any other antibiotic, preferred (green) prescriptions, non-firstline (red) prescriptions. Each OOH received an overview as a quarterly newsletter. GPs could also sign up for bi-monthly text messages with general (generic) short pointers for improvement.

### Statistical analysis

Data was extracted in Excel and analysed using SPSS version 25 (IBM, Armonk, NY). Graphs were generated in Excel and IBM/SPSS. Univariate comparisons (Chi square) were performed to generate a *p*-value of the difference in the percentage of green/red/co-amoxyclav prescribing before and after the intervention.

## Results

Baseline prescribing of antibiotics during this period showed 44% of the prescriptions to be non-firstline (red). The mean percentage of co-amoxyclav, the most prescribed non-firstline prescription, was 33% of all antibiotic prescriptions. The mean percentage of green prescriptions was 56% during this period (Table 2).

**Table 2.** Prescription of green/red/co-amoxyclav prescribing before and after the intervention and *p*-value of comparison before/after (Chi square).

	Before		After		*p* Value
	Mean	SD	Mean	SD	
Percentage Green prescriptions	55.7%	3.1	82.7%	9.8	<0.05
Percentage Red prescriptions	44.4%	3.1	17.1%	6.6	<0.05
Percentage Co-amoxyclav (of total)	32.8%	2.4	10.1%	4.8	<0.05
Total number of prescriptions	16,302		7106		

**Figure 2.**
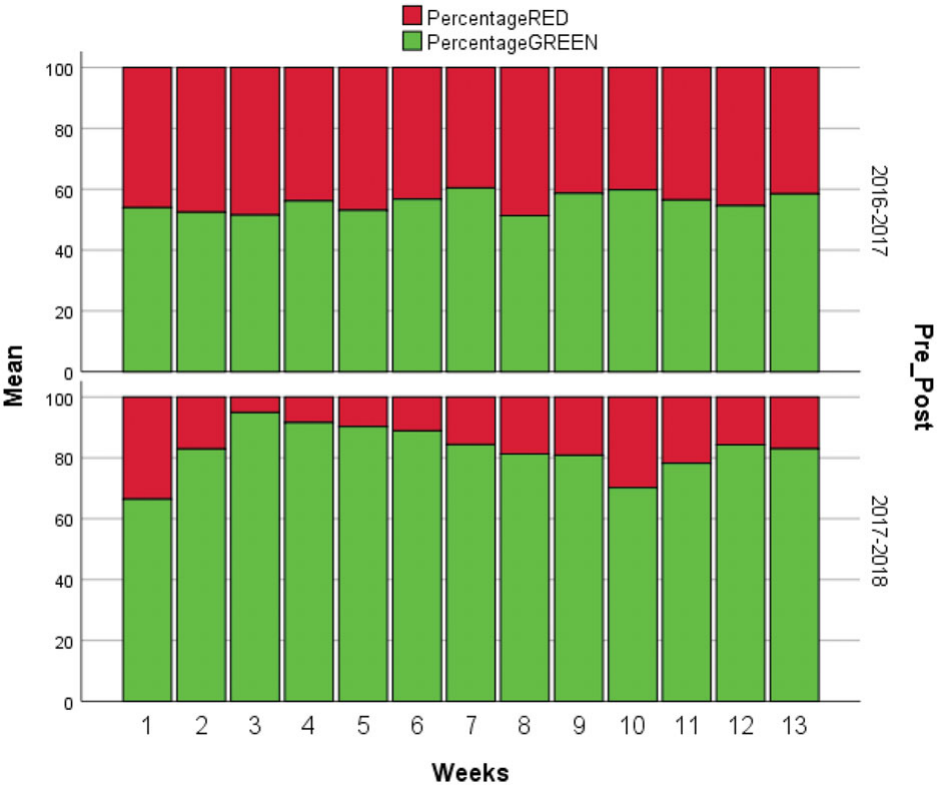
Overview of the mean percentage red and green prescribing in the corresponding weeks before (top panel) and after (bottom panel) the intervention. For the purpose of this figure, the total number of red and green prescriptions was equalled to 100%.

**Figure 3.**
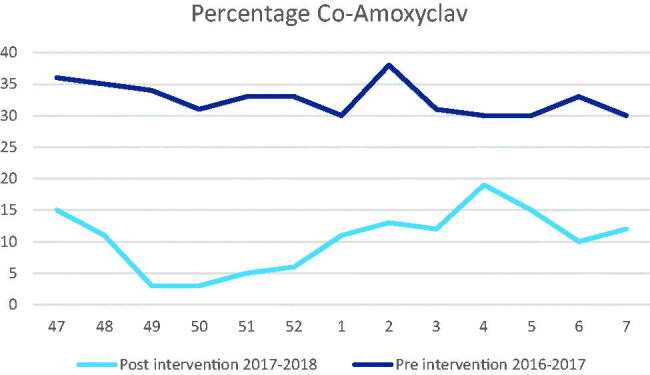
Overview of the pre- and post-intervention co-amoxyclav prescribing as percentage of the total amount of antimicrobials prescribed.

After the intervention was introduced in the OOH services, the percentage was calculated for the same period of the next year (Figures 2 and 3). Percentages dropped for the red prescriptions to 17% and for co-amoxyclav to 10%. An absolute reduction in the percentage of 27.3% and 22.7%, respectively, while the absolute increase of green prescriptions was 27.0%. Table 3 shows more detail of the antibiotic prescriptions, in which a particular increase in amoxicillin and Phenoxymethylpenicillin (Penicillin V) can be observed at the expense of co-amoxyclav.

**Table 3.** Detail of the antibiotic prescriptions before and after the intervention according to the green/red categorisation.

	Before	After
Green antibiotics		
Phenoxymethylpenicillin	0%	10%
Amoxicillin	35%	55%
Flucloxacillin	4%	5%
Trimethoprim	1%	2%
Nitrofurantoin	1%	1%
Red antibiotics		
Co-Amoxyclav	33%	10%
Tetracycline	1%	2%
Macrolide	13%	7%
Cephalosporin	9%	7%
Quinolones	3%	2%
Total number of prescriptions	16,302	7106

## Discussion

### Main findings

The reduction in red prescribing (from 44% to 17%) coupled with the increase of green prescriptions (from 56% to 83%) show that the intervention improved the quality of antibiotic prescribing in OOH. In particular, a reduction in the prescription of co-amoxyclav (from 33% to 10%) was achieved. The categorising of antibiotics into a red and green panel was well received and followed up.

### Interpretation of the results

For Ireland, where co-amoxyclav prescribing is very high, the importance of reducing co-amoxyclav prescribing is of particular importance in relation to the direct link with the occurrence of antimicrobial resistance. In Ireland, resistance levels for co-amoxyclav are nearly 50% for E.coli and 30% for Klebsiella pneumoniae, which are often the cause of common infections. It has been shown that individual exposure to co-amoxyclav is a risk factor for UTIs caused by co-amoxyclav-resistant E. coli or for carrying co-amoxyclav-resistant Gram-negative bacilli in the digestive tract [12]. Our study was set up as a quality improvement intervention and the OOH setting is not conducive to observe AMR. However, the reduction from 33% to 12% co-amoxyclav prescribing can be expected to have an impact on local resistance levels when considering that a reduction in co-amoxyclav prescribing (from 30% to 4%) showed a direct impact on the occurrence of co-amoxyclav resistance in E.coli (from 37% to 11%), within months [13]. Firstline antibiotic recommendations, which are covered under the green prescriptions, are generally selected based on their microbiological activity in combination with tolerance and toxicity. Reducing red prescriptions and co-amoxyclav prescriptions in particular, have the potential to reduce adverse events, when replaced by green prescriptions.

### Strengths and limitations

Our study accomplished a substantial improvement in the quality of antimicrobial prescribing in OOH. Our study is, however, different from most other studies to improve antibiotic prescribing in the OOH setting as the OOH setting was used as an educational platform to reach a larger cohort of GPs rather than to improve prescribing in the actual OOH setting [14,15]. By introducing the educational supports with an automated pop-up as part of the OOH patient management system, potentially more than 200 GPs were reached. Even though GPs will not be reminded by a pop-up when returning to daily duties in their GP practice, the hope would be that the experience with the red/green categorisation will be retained and applied in daily practice.

One of the limitations of our study relates to the OOH setting in which no knock-on effect of the intervention outside this setting could be evaluated nor could the effect of the intervention be measured through a change in resistance levels. The OOH setting has its own patient management system and the pop-up was designed to work within this system. Similarly, any sample taking or its microbiological results would be referred to the daily general practice setting. Also, the intervention was set up to improve the quality of prescribing and as the pop-up appears after the decision to prescribe an antibiotic, no impact on the quantity of antibiotics is expected. However, as the intervention has shown to be successful, opportunities to use the OOH setting to extend the intervention to reduce the quantity of antibiotic prescribing are further explored.

A second limitation is the lack of actual prescription volume. Whereas it is clear quality improved, it is not known if quantity of prescribing remained the same. Again, future analyses of the additional data could evaluate the impact of the intervention on the volume of antimicrobial prescribing in each category.

### Implications

Due to the success of the intervention, the educational material, in particular, the categorisation into red and green antimicrobials, is now freely available on the open-access www.antibioticprescribing.ie website, and the intervention has been expanded to include 23 OOH centres serviced by more than 500 GPs (population coverage 665,000). Meanwhile, the national prescribing database (PCRS-Primary Care Reimbursement Scheme), which includes all prescriptions for medical cardholders (free medical care for patients under an income threshold), has developed a tool to generate a report on antibiotic prescribing for every GP, regularly. This report is now similarly set up with red versus green prescribing. This national database will in time allow to observe and analyse changes in relation to the red/green categorisation.

The intervention is currently being rolled out across all OOH services in this area and the educational material, in particular in relation to the red/green categorisation, is simultaneously being promoted. Whereas literature on antibiotics prescribing in OOH settings has mainly focussed on the difference with daily practice, we used this setting as a more efficient platform to provide GPs with an educational intervention. Depending on the organisation of OOH services in different countries, this efficient strategy could be implemented to improve prescribing of any medication, in particular as no other study was identified using a similar strategy to address inappropriate (antibiotic) prescribing in OOH services.

## Conclusion

A simple and easy to understand categorisation of antibiotics into red (to be avoided) and green (firstline/preferred), in combination with a pop-up message to request details of antimicrobial prescribing in the patient management software of OOH-centres in Ireland, resulted in an absolute reduction of 27% in red prescriptions and 23% in co-amoxyclav prescriptions. Most GPs participate in OOH service and this setting provides an efficient approach to delivering prescribing interventions.
